# Mobility of pectin methylesterase in pectin/cellulose gels is enhanced by the presence of cellulose and by its catalytic capacity

**DOI:** 10.1038/s41598-019-49108-x

**Published:** 2019-08-29

**Authors:** Estelle Bonnin, Camille Alvarado, Marie-Jeanne Crépeau, Brigitte Bouchet, Catherine Garnier, Frédéric Jamme, Marie-Françoise Devaux

**Affiliations:** 1 INRA, UR 1268 Biopolymères – Interactions – Assemblages, 44 316, Nantes, France; 2 Synchrotron SOLEIL, L’Orme des Merisiers, 91190 Saint-Aubin, France

**Keywords:** Hydrolases, Molecular engineering in plants

## Abstract

The pectin methylesterase action is usually studied in a homogeneous aqueous medium in the presence of a large excess of soluble substrate and water. However in the cell wall, the water content is much lower, the substrate is cross-linked with itself or with other polymers, and the enzyme has to diffuse through the solid matrix before catalysing the linkage breakdown. As plant primary cell walls can be considered as cellulose-reinforced hydrogels, this study investigated the diffusion of a fungal pectin methylesterase in pectin/cellulose gels used as cell wall-mimicking matrix to understand the impact of this matrix and its (micro) structure on the enzyme’s diffusion within it. The enzyme mobility was followed by synchrotron microscopy thanks to its auto-fluorescence after deep-UV excitation. Time-lapse imaging and quantification of intensity signal by image analysis revealed that the diffusion of the enzyme was impacted by at least two criteria: (i) only the active enzyme was able to diffuse, showing that the mobility was related to the catalytic ability, and (ii) the diffusion was improved by the presence of cellulose in the gel.

## Introduction

Plant cell walls consist mainly of polymer assemblies (pectin, hemicelluloses, cellulose and proteins), whose structure and interactions vary depending on plant genetics and development. Cellulose is a homopolymer of β (1–4) glucose most often considered as a load-bearing component in the primary cell wall. Pectin is a chemically more complex polysaccharide characterized by a high content in α (1–4) galacturonic acid (GalA) that may carry a methyl ester. Pectin is able to interact with divalent cations such as calcium to form a physical gel set up *via* electrostatic interactions involving the free carboxylate groups of GalA. Since 8–15 consecutive non-methyl esterified GalA units are required to form a stable calcium-mediated junction zone^1,2^, the distribution of the non-methyl esterified residues is a key parameter for predicting the gelation ability of pectin^3–7^. Therefore, pectin ability to gel is favoured by the action of pectin methylesterases (PME), which de-esterify GalA carrying methyl ester groups and belong to the CE8 family of the Carbohydrate Esterases (www.cazy.org). When a sufficient number of contiguous free GalA residues is generated by enzyme action, new junction zones can be established between pectin chains in the presence of calcium. As a consequence, the gelling ability of enzymatically-deesterified pectin will depend on the mode of action of the PME, and more precisely on its processivity. Processivity, first described for several α-amylases^8,9^ refers to as the cleavage of several adjacent linkages without dissociation of the enzyme from its substrate after each catalytic event. When studied on a soluble substrate and in very dilute conditions, the mode of action of PME was shown to be dependant on their origin. Fungal PME release short sequences of free GalA residues, following a multiple-chain and multiple-attack mechanism^3,10,11^. On the contrary, plant PME act according to a single-chain and multiple attack mechanism, thus behaving as a processive enzyme and releasing blockwise distributed free GalA^4,12–15^. In all these studies, the modes of action were determined on a soluble substrate in dilute solutions, *i.e*. in a large excess of substrate and in homogenous medium. These conditions are far from those met in the cell wall, where the dry matter is much higher (30%^16^), the substrate is cross-linked with itself or with other polymers, and the enzyme has to reach its substrate by diffusing through the solid matrix before catalysing the linkage breakdown. Thereby, it is likely that biochemical properties established *in vitro* do not reflect the enzyme mechanism occurring *in muro*. As the complexity of the cell wall and its variability make difficult to follow the enzyme action *in muro*, we chose to use model systems mimicking the cell wall. To investigate the behaviour of PME, we first used pectin-calcium gels as the substrate^7,17,18^. The particularity of this system is that the gel strength increases during the gelation kinetics since the enzyme releases new sites of interactions with calcium. Compared to their mechanism in pectin solution, the processivity of fungal PME was reinforced and the one of plant PME was diminished when they act on a pectin-calcium gel^7,19^. The presence of calcium would generate physical constraints that would affect the catalytic properties of the enzyme^17^ and hamper its mobility^18^. As the cell wall is much more complex than a pectin gel, we then used pectin/cellulose gels to study the PME mechanism^20^. Mixing cellulose and pectin in the presence of calcium led to a composite and interpenetrated gel where the two polysaccharides are however localized in different phases^21^. The gel seemed to adopt different microstructures according to the respective proportions of the two polysaccharides^20^. When increasing the proportion of cellulose, a fraction of pectin would form close interaction with the microfibrils. This would result in a larger volume accessible to diffusing enzyme and in an accelerated kinetics for the fungal PME. However, these results were obtained at a macro-scale, in gels of 2 cm high and 1.4 cm diameter, which did not allow investigating the accessibility of the pectin to the PME in relation to the presence of cellulose, and the way the PME diffuses in the gel.

The fluorescence techniques used to date allowed decreasing the distance- and the time-scale of observation^18^ but required the use of fluorescent labelled enzyme that may affect its activity or specificity by inducing steric constraints hampering protein flexibility. To avoid labelling, it is possible to exploit the auto-fluorescence of tryptophan and tyrosine after excitation in deep UV^22,23^. Indeed, it was previously demonstrated that excitation at 275 nm allowed imaging two different amylases, including their adsorption and diffusion on and within starch granules at a not previously attained spatial resolution^24^. More recently, enzymatic degradation of maize stem was followed by multichannel auto-fluorescence imaging of a cellulase cocktail and the cell wall phenolic compounds^25^. Both experiments were carried out on the low-energy beamline DISCO at the French synchrotron SOLEIL on a full-field fluorescence microscope, and gave the opportunity to follow unlabelled enzymes at high resolution and in real time. From this background, we aim to investigate the PME diffusion in a cell wall-mimicking matrix and to understand the impact of this matrix and its (micro) structure on the efficiency and the mode of action of the enzyme. As we demonstrated previously that the PME from *Aspergillus aculeatus* diffused faster than the orange PME, the fungal PME was applied on pectin/cellulose gels prepared with increasing contents in cellulose. The mobility of the enzyme was recorded to understand how it diffuses with respect to the matrix structure, if the physical constraints entailed by the solid state of the matrix impact the enzyme behaviour, and if the gel reinforcement impacts the distribution of the cellulose chains within the gel. It was also recorded for active and inactivated enzyme to investigate the impact of catalysis on the enzyme mobility.

## Experimental

### Polysaccharides

The citrus pectin was kindly provided by Cargill Texturizing Solutions (lot n° 12030618, Baupte, France). It contains 85.07% GalA and has a degree of methylation of 46^20^. It is therefore qualified as low methylated pectin and noted LM pectin.

Cellulose microfibrils were extracted from sugar beet pulp^26^ and contained 80.3% glucose and 8.1% of other neutral sugars and uronic acids. Defrosted cellulose suspension at 1% w/w was sonicated twice for 4 min in a Branson 200 ultrasonic cleaner, and then concentrated to 15% w/w by osmotic compression dialysis against dextran solution (18% w/w, Mw = 100,000 g/mol).

### Gel preparation

**Figure 1:**
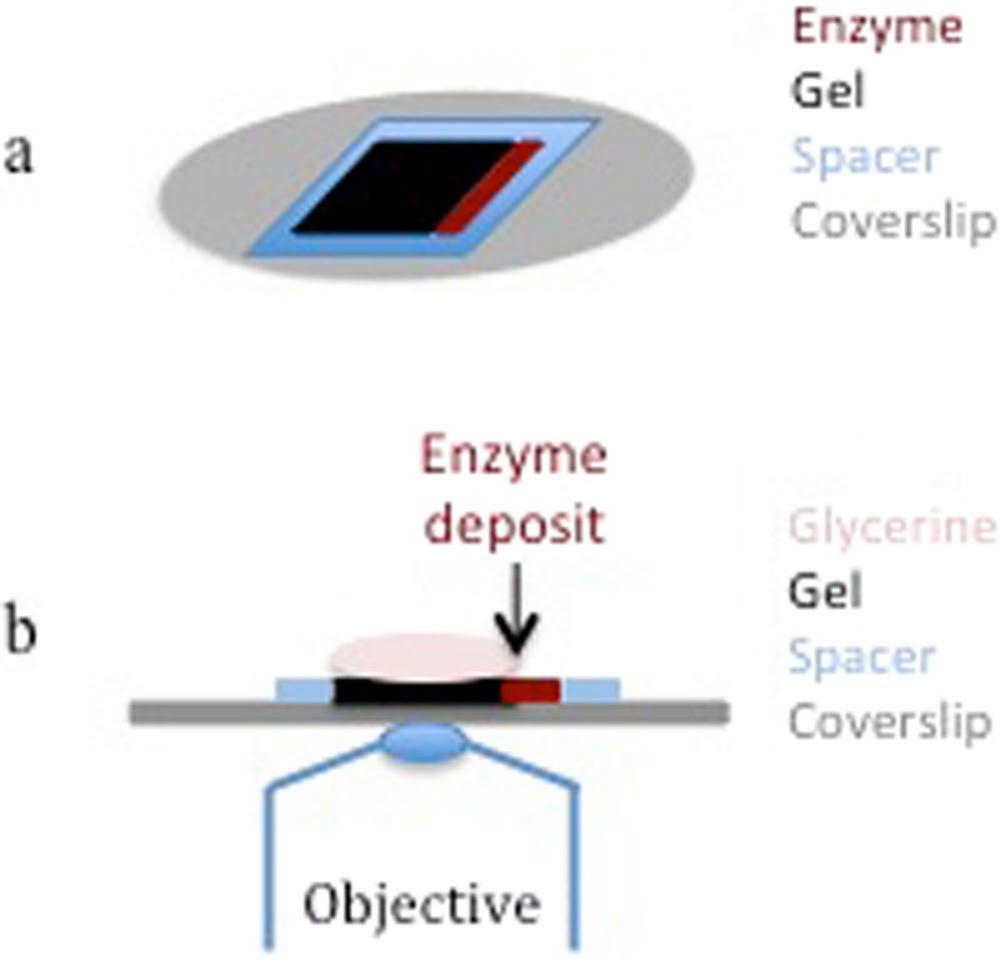
Sample presentation. (**a**) Schematic perspective view of the sample presentation: the gel (black) was poured in a 10 × 10 mm spacer (blue) on the coverslip (gray). The enzyme (maroon) was deposited on the right side of the gel. The sample was covered with glycerine before observation (not represented). (**b**) Schematic side view of the gel (black) poured in a spacer (blue) on the coverslip (gray), and then covered with glycerine (light pink) and observed on the inverted microscope. The enzyme (maroon) was deposited on the right side of the gel

To evaluate the effect of the ratio between the two polysaccharides on the enzyme diffusion, the binary gels were prepared by mixing LM pectin at a fixed concentration (1.5% final concentration) with an increasing concentration of cellulose (from 0 to 1.5% final concentration). To ensure the gelation of the mixtures, calcium chloride was added at 5 mM final concentration as previously described^20^. The pectin/cellulose mixtures were extemporaneously melted before being poured on a coverslip (A1 fused quartz R525000, ESCO OPTICS, New Jersey, USA) to allow the gelation just before testing the diffusion. The gels were referred to as g00 (0% cellulose), g05 (0.5% cellulose), g10 (1.0% cellulose) and g15 (1.5% cellulose). They were poured in a square delimited by a frame of 250 µm thickness (hybridation frame 25 µL, 10 × 10 mm, AB-0576 ThermoFisher) and a channel was arranged on the right side of the spacer to deposit the enzyme (Fig. 1a). To avoid gel desiccation, the gel was left in a wet box during 1 h gelation at room temperature and covered with glycerine (Carl Zeiss GmbH, Germany) after enzyme deposit and before observation.

### Enzyme

The PME used in this study was from *Aspergillus aculeatus* (APME, EC 3.1.1.11, UniProt Q12535) and kindly provided by Novozyme A/S (Copenhaguen, Denmark). The processed APME contains 314 amino acids (331 in the full length protein – 17 in the peptide signal), including 6 tryptophan and 21 tyrosine residues^27^. It was prepared at 67.5 mg/mL in 50 mM MES buffer pH 6. Its activity was checked in these conditions^17^. The required activity was calculated according to the degree of methylation of the pectin and the concentration of the pectin solution, and was fixed at 0.6 U/sample, which corresponded to 1 µL of APME solution. Part of the solution was inactivated in a boiling water bath. Time course of de-esterification experiments were conducted with native APME (APMEa) and with the inactivated enzyme (APMEi) in the same conditions.

### Synchrotron deep UV fluorescence imaging

#### Acquisition parameters

The full field microscope TELEMOS used to follow enzyme diffusion on the DISCO beamline is an inverted microscope modified from a Axio Observer Z1 (Carl Zeiss GmbH, Germany)^22,23,28^ controlled by MicroManager software (https://micro-manager.org). The synchrotron beam was monochromatized by an iHR320 monochromator (Jobin-Yvon Horiba, Longjumeau, France). The excitation wavelength was set at 275 nm to recover the auto-fluorescence from tyrosine and tryptophan. The objective was an Ultrafluar 40 × (Carl Zeiss GmbH, Germany; numerical aperture 0.6) leading to of field of view of 287 × 287 µm2 with pixel size of 0.2804 µm. It was covered with glycerine before sample positioning. Fluorescence images were acquired through a dichroic mirror at 300 nm (Omega Optical Inc., USA) and a single bandpass filter 340/26 nm (Semrock, Rochester, USA) and recorded using a back-illuminated CCD camera (PIXIS 1024B/BUV, Princeton Instruments, USA) that codes the fluorescence intensity in 2^16^ = 65536 gray levels. In order to capture all the information of the microscope into the image (Nyquist criterion) the ideal lateral sampling has been estimated at 0.22 µm and axial sampling at 1 µm. By using a pixel size of 0.28 µm we are slightly under sampling condition.

To recover sufficient auto-fluorescence intensity from tryptophan and tyrosine on the whole field of the x40 objective, the recording time was set at 7 s per image. In these conditions, the absence of UV fluorescence was checked in the binary gels before enzyme deposit.

#### Sample presentation

Since the microscope installed on the DISCO beamline is an inverted microscope, the observable region of the sample is just above the coverslip (Fig. 1b). The enzyme was deposited in the channel arranged on the right side of the gel when pouring to ensure that the enzyme was present only on one side of the gel and followed a unidirectional diffusion. After focusing, images were acquired over time.

#### Regions Of Interest

**Figure 2:**
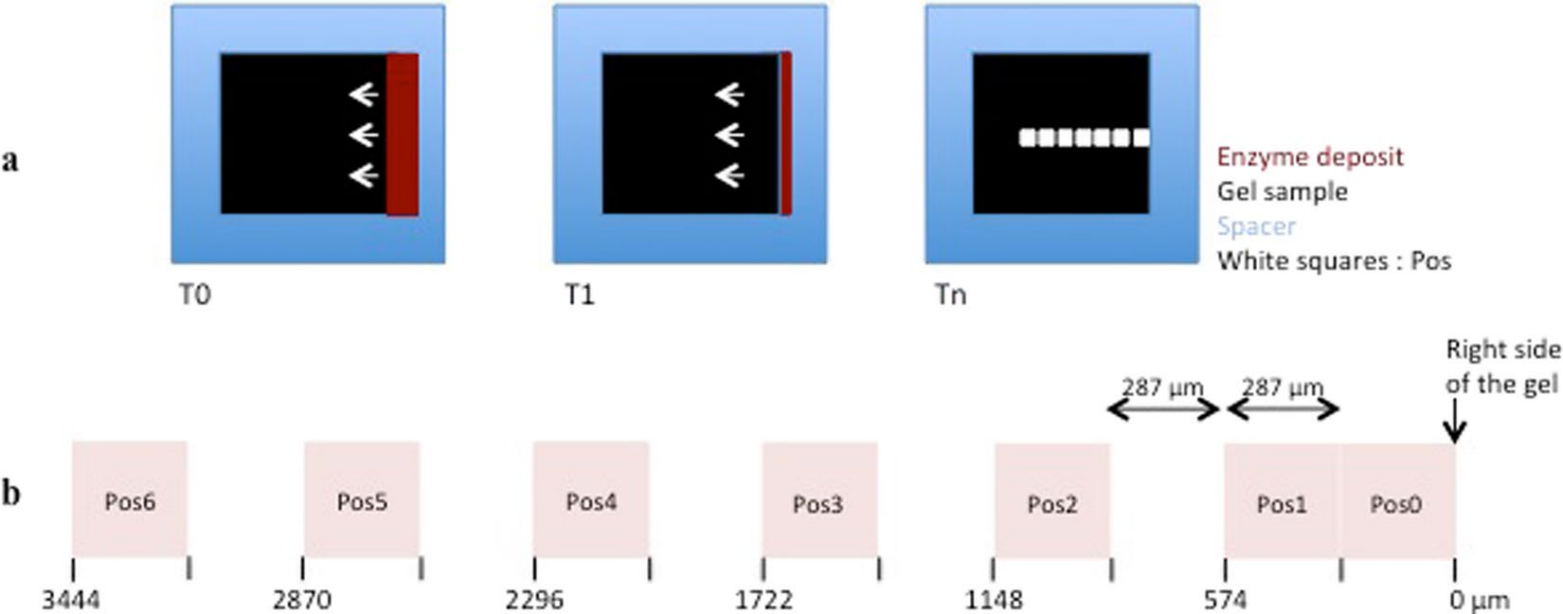
Definition of the regions of interest. (**a**) Schematic top view of enzyme diffusion (maroon) inside the gel sample (black) and definition of the regions of interest, referred to as Pos for Position (white squares). Three time points are represented: at T0, the channel is full of enzyme (maroon); at T1, the enzyme diffuses into the gel according to the white arrows; at Tn, the channel is empty. (**b**) From the deposit point of the enzyme (right side of the gel), the 2 first Pos corresponded to the two first fields at magnification x40, the next ones corresponded to 1 field over 2. The 7 positions were numbered from Pos0 to Pos6. Values below the positions are the distance from the deposit point.

From the right side of the gel, 7 regions of interest were selected along the migration of the enzyme (Fig. 2a). They were abbreviated as Pos, for Position. As shown on Fig. 2b, the two first Pos were contiguous, while the following were spaced by the width of one field. Z stacks of 5 images were acquired on each Pos with a range of 1 µm (−2 µm/+2 µm).

#### Time-lapse acquisition

Images of the 7 Pos (called from Pos0 to Pos6) were acquired during time course experiments. The time interval between two acquisitions was 5 min. Twelve time points were acquired for each time course experiment during 55 min. The time course experiments were carried out in duplicate.

### Quantification of intensities by image analysis

Fluorescence intensity was considered as representative of enzyme amount along the time course. The fluorescence intensities were extracted from the images to draw kinetic curves. The successive steps were: image pre-processing, intensity measurement on pixel lines, calculation of the average intensity of selected pixels, plotting of time and spatial kinetics of intensity evolution.

#### Image pre-processing

All the fluorescence images were pre-processed to remove the camera-related background and compensate for the illumination inhomogeneity using the principles previously described^29^ for shading correction:

1 $${\rm{IMC}}=(\mathrm{IM}-\mathrm{BKG})/\mathrm{ILL}$$

where IM is the raw image, BKG is the additive background, ILL is the illumination inhomogeneity, and IMC is the corrected image. Several black camera images were recorded and averaged to measure the background BKG. The illumination inhomogeneity was estimated for each experiment from raw image at position Pos0. The following filters were successively applied: morphological opening, morphological closing and average filter. The filter size was 105 pixels for all^30^.

#### Intensity measurements

Several ways were tested to extract intensity values from the images. The intensity was measured on circles corresponding to 1/2 or 3/4 of the width of the image, or on a random line. The random line had the advantage of avoiding cellulose particles when they were present. Thus, for each image, *i.e*. each Pos of each experiment, a set of pixels was selected by drawing a random line in the field. The fluorescence intensity was measured and averaged over z. The profiles I = f (Pos, t) were extracted for each experiment.

#### Time and spatial evolution of fluorescence intensity

Intensities were plotted as a function of time or position for active and inactive PME. In order to take into account small variations in the fluorescence signal at Pos0, kinetics were normalized by dividing all the intensity values by the intensity observed at Pos0.

Image processing were performed using the image processing and statistics and machine learning toolboxes from MATLAB (2015) (The MathWorks, France, available on line at https://fr.mathworks.com/products/new_products/release2015a.html).

## Results

### Microscopic structure of cellulose-pectin gels prepared with varying concentrations of cellulose

**Figure 3:**
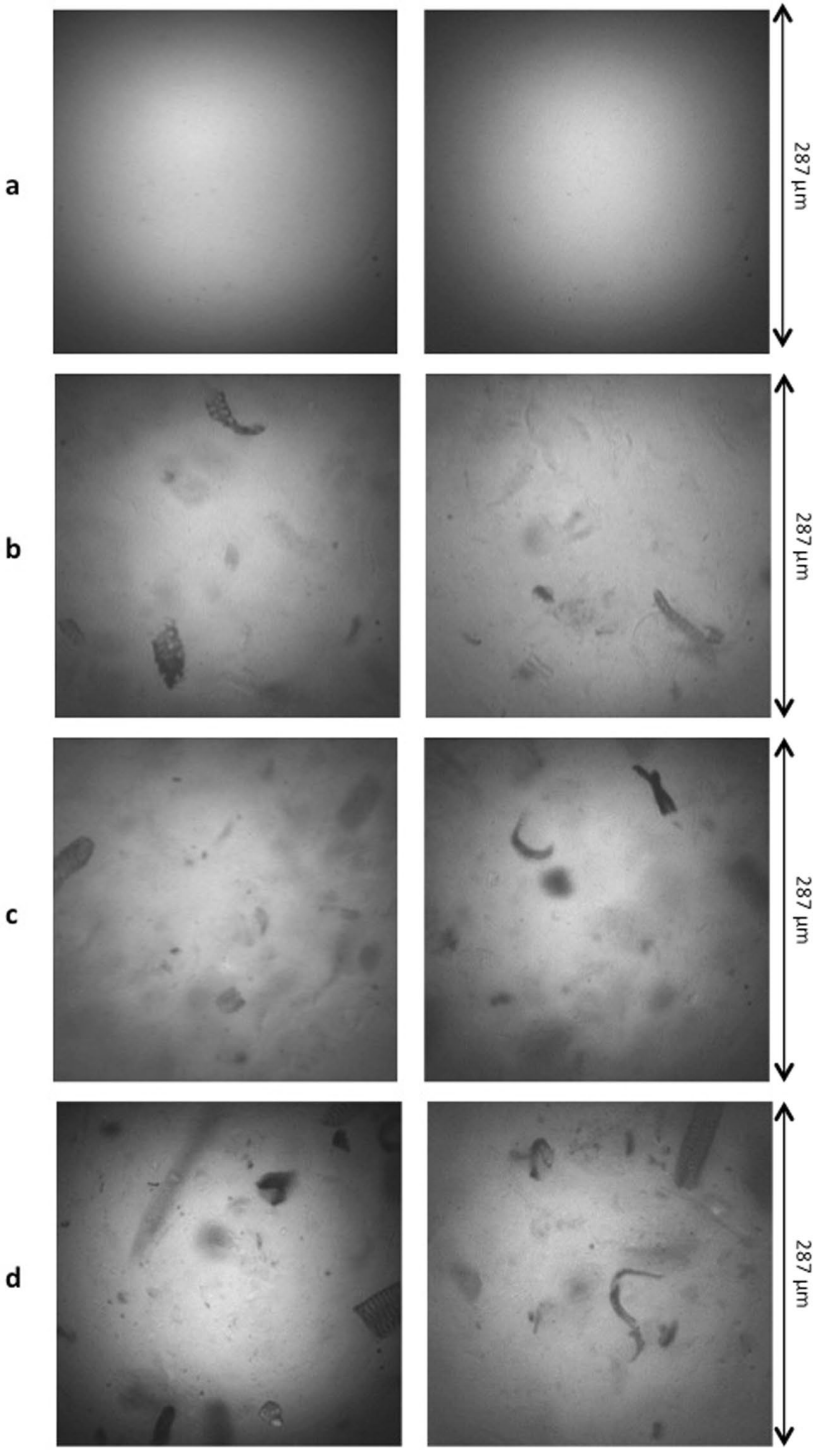
Raw fluorescence images of Pos0 at time T0 of two kinetics carried out on each gel type: (**a**), 0% cellulose; (**b**), 0.5% cellulose; (**c**), 1.0% cellulose; (**d**), 1.5% cellulose. To allow comparison, brightness and contrast were similarly adjusted for all images. The full field of view was 287 × 287 µm^2^.

Gels were prepared with a fixed concentration of LM pectin (1.5%) and varying concentrations of cellulose, 0.0%, 0.5%, 1.0% and 1.5%. This resulted in water content in the gels varying from 97% to 99.5%. During enzymatic de-esterification using APME, the enzyme was followed *in situ* by recording the auto-fluorescence of its aromatic amino acids after excitation at 275 nm using the synchrotron beam. As soon as the enzyme was deposited on the gel, fluorescence images were acquired at Pos0 and raw images are shown on Fig. 3. As the absence of fluorescent background was checked before enzyme deposit, the fluorescence collected with the 340/26 nm filter was fully ascribed to the enzyme APME. In the cellulose-free gel (referred to as g00), the enzyme-related fluorescence was evenly distributed over the image suggesting a homogenous gelation of the LM pectin and a homogenous distribution of APME on its substrate. On the contrary adding cellulose in the gel at 0.5% (g05), 1.0% (g10) or 1.5% (g15) lead to a major change of the image texture. Non-fluorescent particles with different sizes and shapes were visible in a continuous fluorescent phase. They likely corresponded to cellulose particles embedded in the pectin network. The enzyme was excluded from the particles and thus showed no affinity for them, as expected. As the pectin used were commercial citrus pectin, it was extracted in harsh acidic conditions and therefore contained only 8.1% of neutral sugars. Thus, it was unlikely that they bind directly to cellulose through the neutral sugar side chains^31^. Moreover, the binary gels were prepared in the presence of calcium, which resulted in a weak pectin/Ca^2+^ gel and in an interpenetrating network of cellulose and pectin. Such cellulose/pectin gels were previously studied by confocal laser scanning microscopy after preparing mixtures with different concentrations of the two polysaccharides and in the presence or not of calcium^21,32^. The use of confocal laser scanning microscopy required labelling one or the other of the two polysaccharides. At this scale, the two polymers were evidenced in separate phases of the mixtures.

### Kinetics of enzymatic de-esterification with APME

**Figure 4:**
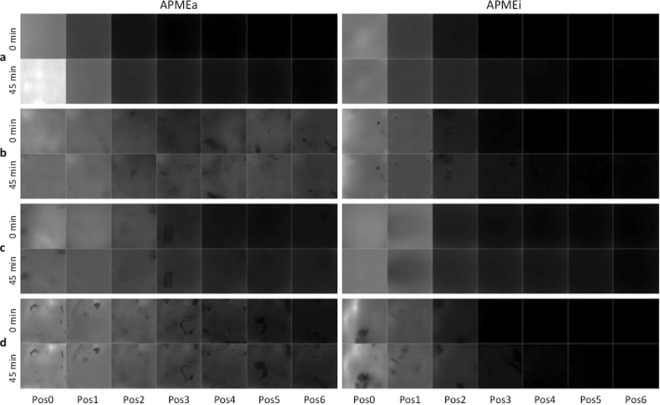
Treated fluorescence images acquired at 0 min and 45 min on all positions (Pos0 to Pos6) for the native (APMEa) and the heat-inactivated (APMEi) enzyme. Gels were prepared by mixing 1.5% LM pectin with (**a**), 0% cellulose; (**b**), 0.5% cellulose; (**c**), 1.0% cellulose; (**d**), 1.5% cellulose. The full field of view was 287 × 287 µm^2^.

Fluorescence images were acquired every 5 min from the moment the enzyme was deposited and for 55 min, corresponding to 12 acquisition sets. Intensities were measured at each time point after image pre-processing. For all experiments, the initial intensities (T0) ranged between 600 and 800 counts. This range may be due to slight variations in enzyme deposit or focal plane setting. To avoid differences in starting values and help compare images, the intensities were normalized on the values at Pos0 and T0 for each kinetic experiment. In Fig. 4, the images acquired at 0 min and 45 min and treated according to this process are presented for the native as well as the heat-inactivated enzyme. The visual comparisons of the images lead to some findings. For the active APME (APMEa) on the cellulose-free gel (Fig. 4a, left), at T0 the enzyme concentrated at Pos0, *i.e*. around the deposit point and started diffusing to Pos1. After 45 min, the enzyme diffused from the deposit point into the gel, and thus the amount largely increased at Pos0 and to a lesser extent at Pos1. A slight signal was still visible at Pos2. When cellulose was present in the gel (Fig. 4b–d left), the active enzyme was much more mobile as it was already present in the distant positions from the beginning of incubation. At T0 with 1% cellulose (Fig. 4c), fluorescence signal was detectable up to Pos3, and up to Pos4 in the presence of 1.5% cellulose (Fig. 4d). When it contained 0.5% cellulose (Fig. 4b) a signal was visible on all the positions. The comparison of starting images and 45 min images indicated that particles visible in Pos4 to Pos6 were slightly displaced, suggesting that the sample moved during observation. In the presence of 1.5% cellulose at 45 min, the signal was detected up to the furthest position resulting from the enzyme mobility. Moreover, it is noteworthy that the position of the particles did not change between 0 and 45 min, suggesting that the strengthening of the gel linked to the enzymatic de-esterification by APME did not exclude them from the gel structure.

The same kinetic experiments were carried out with the inactivated enzyme (APMEi, Fig. 4 right). Thermal inactivation of the enzyme was expected to induce unfolding of the enzyme. The images acquired with APMEi were almost all identical. The fluorescence signal did not exceed Pos2 whatever the cellulose content in the gel and the time of observation, showing that the inactive enzyme did not diffuse.

**Figure 5:**
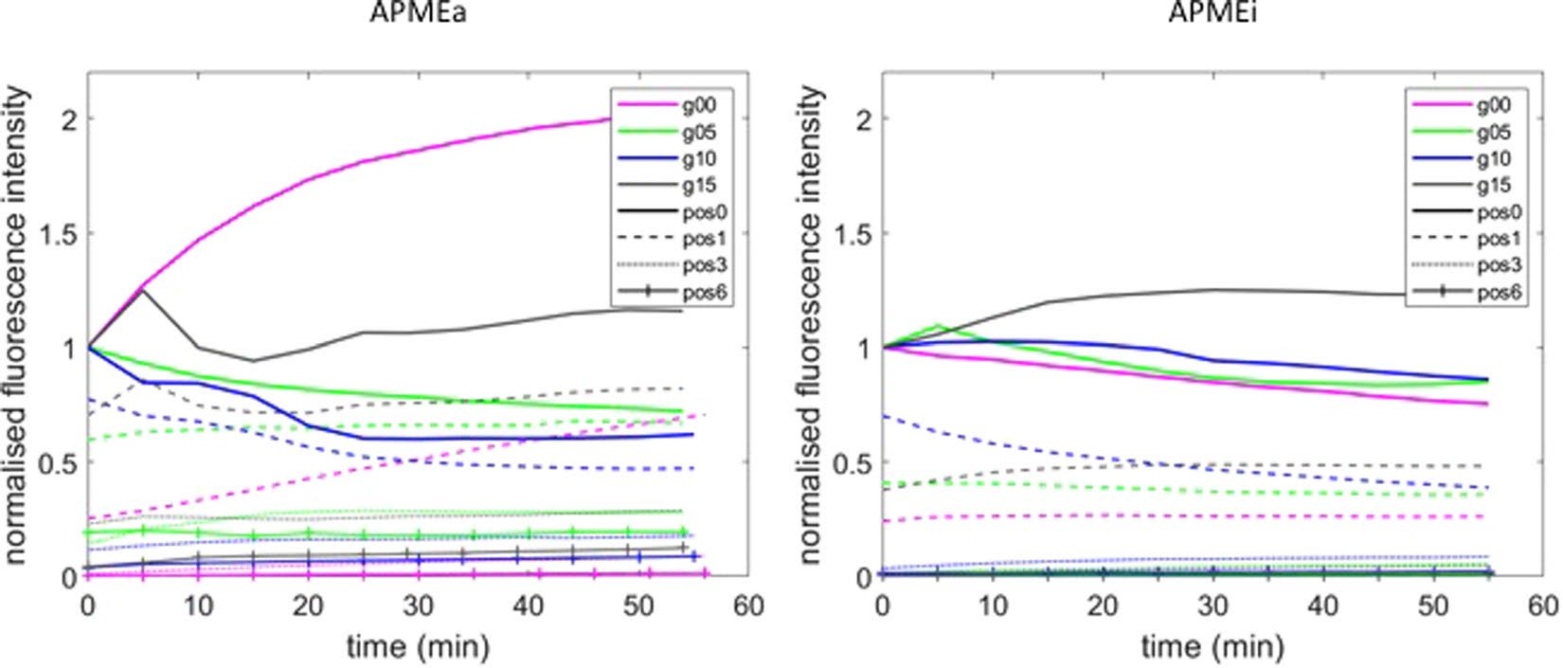
Evolution of fluorescence intensity as a function of time. The fluorescent intensities were extracted from images acquired during 55 min on positions 0 (Pos0), position 1 (Pos1), position 3 (Pos3) and position 6 (Pos6) for the native (APMEa) and the heat-inactivated (APMEi) enzyme. The intensity values were normalized on the values at Pos0 and T0 for each kinetic experiment. The gels were prepared by mixing 1.5% LM pectin with no cellulose (g00, pink), 0.5% cellulose (g05, green), 1.0% cellulose (g10, blue), 1.5% cellulose (g15, black).

To analyse in more details, fluorescence intensity values were extracted from all the images. From previous observations and image analysis^25^, the fluorescence intensities recovered with the 340/26 nm filter were assumed to be proportional to the enzyme amount. In Fig. 5, the extracted values were plotted as a function of time for the active and the inactive enzymes. To simplify, only the values from Pos0, Pos1, Pos3 and Pos6 are shown. Due to the normalization of each kinetic set on the intensity at Pos0, the curves of all Pos0 started at 1. With active APME, only the intensity of Pos0 in cellulose-free gel (g00) increased, with a slope becoming weaker during the course of de-esterification. For the other gels, the intensity of Pos0 slightly decreased (0.5% and 1.0% cellulose) or kept rather constant (1.5% cellulose). The intensity increased slowly and steadily at Pos1 of cellulose-free gel whereas it kept rather constant for the cellulose-containing gels. For the other positions, fluorescence intensity remained low and did not change with time. The intensity was nevertheless higher for the cellulose-containing gels compared to the cellulose-free gel. Although the enzyme seemed to be migrating further on the gel with 0.5% cellulose (Fig. 4b), the extracted values suggested the same tendency as the values extracted from the other gels. With the inactive enzyme APMEi, it appeared clearly that the diffusion was similar in all gels. In all cases, intensity at Pos1 was about half of that at Pos0, a slight signal was still visible on Pos2 (Fig. 4) and was null on the more distant positions.

**Figure 6:**
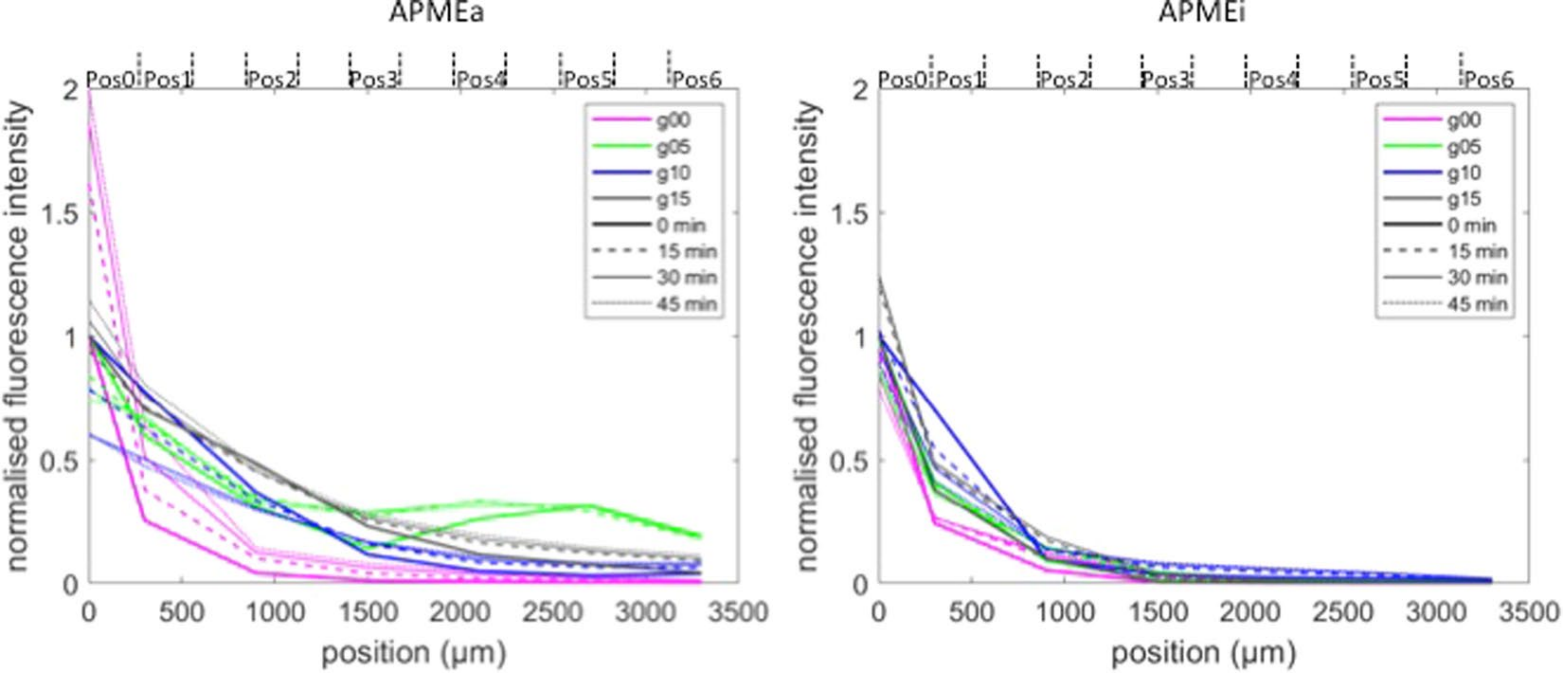
Evolution of fluorescence intensity as a function of Position. The fluorescent intensities were extracted from images acquired on all positions at 0, 15, 30 and 45 min for the native (APMEa) and the heat-inactivated (APMEi) enzyme. The intensity values were normalized on the values at Pos0 and T0 for each kinetic experiment. The gels were prepared by mixing 1.5% LM pectin with no cellulose (g00, pink), 0.5% cellulose (g05, green), 1.0% cellulose (g10, blue), 1.5% cellulose (g15, black).

To show the enzyme diffusion in space, the variations of the intensity were plotted as a function of the positions for different incubation times (Fig. 6). Again, the normalized values of intensity were used and 4 incubation times were plotted: 0, 15 min, 30 min and 45 min. This allows comparing the enzyme diffusion in the 4 different gels for both the active and the inactivated enzymes. The graph drawn for the active enzyme (Fig. 6 left) particularly highlights that at T0 in the gel 00, the fluorescence of the active enzyme fell to its threshold at Pos2, starting at 860 μm, whereas in the other gels, the fluorescence signal reached its threshold at Pos3 or Pos4, around 1500–2000 µm (see Fig. 2 for the distances). After incubation, the fluorescence intensity at Pos0 in the cellulose-free gel (g00) largely increased (x 2 after 45 min) and little increased at more distant positions. In the opposite in cellulose-containing gels, intensity tended to decrease at Pos0 and the enzyme seemed to diffuse further with time. Again, these curves show that the enzyme diffused further in the presence of cellulose. The most favourable conditions for the diffusion of the active enzyme seemed to be the gel containing 0.5% cellulose (g05). The curve reflects an unexplained behaviour at positions 4, 5 and 6, where the enzyme was unexpectedly high. The curves obtained before normalization of the values showed the same phenomenon, demonstrating that this was not caused by normalization (Supplementary Data). With the inactivated enzyme (Fig. 6 right), the curves drawn for the four gel types superimpose, showing that the enzyme did not diffuse with time. One exception was the Pos1 on the gel containing 1% cellulose (g10), where the curve at T0 shows the presence of enzyme, as already observed on Fig. 4c left.

## Discussion

At the microscopic level, transport by diffusion can be defined as a passive transport due to Brownian motion. It is due to the presence of a gradient of concentration of the diffusing species and tends to homogenise the medium. Therefore, the diffusion results in a return to the thermodynamic equilibrium. The main theories for diffusion were previously reviewed^33,34^ and indicate that polymer chains in gel slow down solute diffusion either by reducing its mean free space because of an increased hydrodynamic volume of the polymer, or by increasing its path length due to obstacles. However, these theories are applicable when the diffusing molecule does not interact with the medium. In the present study, APMEa is specific of one of the polysaccharides involved in the gel. The enzyme action has two different consequences: first, the enzyme binds the pectin chain according to a more or less processive binding mode^17^, and second, it produces new free carboxylate groups in pectin. This second phenomenon allowed the formation of junction zones strengthening the gel during the incubation and the diffusion of the enzyme^7^. On the contrary, the inactivated enzyme APMEi can be regarded as a passive molecule that does not interact with the polymers present in the gel.

Taken together, our results suggest that at least two criteria conditioned the diffusion behaviour of the enzyme in the gel. One was related to the gel structure, the other one was the catalysis ability of the enzyme.

In a pectin/calcium gel, calcium only interacts with carboxylate groups^35^. As a consequence, Ca^2+^ cations form ionic bridges between two galacturonates. Several calcium bridges on adjacent galacturonates form junction zones, lead to a loose association between pectin chains and to a rather homogeneous network of pectin. When cellulose is added before forming gels, gelation of the LM pectin occurred in the presence of cellulose and provided a composite gel. Rheological measurements showed that the storage modulus of pectin/cellulose gels increased with the cellulose concentration in the mixture^21^. The porosity of these composite gels was investigated by following the diffusion of two pullulans having hydrodynamic volumes of 5 to 8 nm^20^. The diffusion of these molecules in a 1.5% pectin gel indicated that the pore size was at least of 8 nm. Adding cellulose up to 1.5% favoured the diffusion of the same species, suggesting that a fraction of pectin would interact with the microfibrils resulting in a larger volume accessible to diffusing molecules. This volume would be related to the cellulose concentration. Conversely, using arabinoxylan and cellulose nanocrystals gels, it has been shown that cellulose nanocrystals do not increase entanglements in the gel, and thus do not impact the diffusion coefficients of diffusing molecules with diameters from 4.6 nm to 22.2 nm^36^. However, the usual length of cellulose nanocrystals is 100–200 nm^37^ whereas the cellulose microfibrils used here were around 10 µm long^26^. It is likely that this huge difference in the length of the two types of cellulose particles explains their respective impacts on the gel properties.

Regarding the PME, it is possible to calculate its diameter using two formula given for the radius of globular proteins as a function of the number of amino acids^38^:

$${\rm{For}}\,{\rm{native}}\,\mathrm{protein},\,{\rm{Rh}}\,({\rm{\AA }})={\rm{4.75}}\times {{\rm{N}}}^{{\rm{0.29}}}$$

$${\rm{For}}\,{\rm{denatured}}\,\mathrm{protein},\,{\rm{Rh}}\,({\rm{\AA }})={\rm{2.21}}\times {{\rm{N}}}^{{\rm{0.57}}}$$

where R_h_ is the hydrodynamic radius, expressed in Angström, and N is the number of amino acids. Taking into account the 314 amino acids present in APME sequence, the diameter of the native enzyme is 5 nm, whereas the diameter of the denatured enzyme is 10.2 nm. The denatured enzyme behaved rather similarly to the pullulans of similar size (Fig. 6 and^20^), meaning that it diffused like a passive molecule. On the contrary, the active enzyme exhibited a much higher mobility, even increasing in the presence of cellulose. Mobility was first considered as independent of enzyme activity. However, it was recently demonstrated that catalysis boosts the motion of enzymes to be superdiffusive for a few microseconds, enhancing their effective diffusivity over longer timescales^39^. Moreover, the boosts would be more frequent at high concentrations in substrates, biasing the enzyme to substrate-poor regions. Fluorescence correlation spectroscopy experiments carried out on enzymes following Michaelis-Menten kinetics suggested that the energy necessary for this motion would come from the heat released during the catalysis^40^. As a consequence, this effect would be the highest for enzymes whose turnover rate is high, and is conversely absent for the inactivated enzyme.

## Conclusion

The present work shows that the PME mobility in a pectin/cellulose gel was catalytically induced and was improved by the presence of cellulose in the gel. As primary cell walls in plant can be considered as cellulose-reinforced hydrogels, these observations made in a model system remain true in the plant cell wall. Indeed the endogenous plant PMEs modulate the degree of methylation in the cell wall and are a key actor in the cell development^41^. On the other hand, the PMEs from phytopathogenic fungi demethylate pectin to promote the action of the fungal polygalacturonase and help the colonization of the host plant.

Furthermore, the work shows the great potential of real-time imaging of unlabelled enzymes for studying the impact of local biochemical variations on their binding and the resulting time-lapse progression^25,42–44^.

## Supplementary information

Supplementary dataset 1

## References

[1] Chen EMW, Mort AJ (1996). Nature of sites hydrolyzable by endopolygalacturonase in partially-esterified homogalacturonans. Carbohydr. Polym..

[2] Benen JAE, Kester HCM, Visser J (1999). Kinetic characterization of *Aspergillus niger* N400 endopolygalacturonases I, II and C. Eur. J. Biochem..

[3] Thibault J-F, Rinaudo M (1985). Interactions of mono- and divalent counterions with alkali- and enzyme-deesterified pectins in salt-free solutions. Biopolymers.

[4] Ralet M-C, Dronnet V, Buchholt HC, Thibault J-F (2001). Enzymatically and chemically de-esterified lime pectins: characterisation, polyelectrolyte behaviour and calcium binding properties. Carbohydr. Res..

[5] Ström A (2007). Influence of pectin fine structure on the mechanical properties of Calcium-pectin and acid-pectin gels. Biomacromolecules.

[6] Löfgren C, Guillotin SE, Evenbratt H, Schols HA, Hermnasson A-M (2005). Effects of calcium, pH, and blockiness on kinetic rheological behavior and microstructure of HM pectin gels. Biomacromolecules.

[7] Slavov A (2009). Gelation of high methoxy pectin in the presence of pectin methylesterases and calcium. Carbohydr. Polym..

[8] Robyt JF, French D (1967). Multiple attack hypothesis of α-amylase action: action of porcine pancreatic, human salivary, and *Aspergillus oryzae* α-amylases. Arch. Biochem. Biophys..

[9] Robyt JF, French D (1970). Multiple attack and polarity of action of porcine pancreatic α-amylase. Arch. Biochem. Biophys..

[10] Limberg G (2000). Analysis of different de-esterification mechanisms for pectin by enzymatic fingerprinting using endopectin lyase and endopolygalacturonase II from *A. niger*. Carbohydr. Res..

[11] Ralet M-C, Thibault J-F (2002). Interchain heterogeneity of enzymatically deesterified lime pectins. Biomacromolecules.

[12] Catoire L, Pierron M, Morvan C, Hervé du Penhoat C, Goldberg R (1998). Investigation of the action patterns of pectinmethylesterase isoforms through kinetic analyses and nmr spectroscopy. J. Biol. Chem..

[13] Denès J-M, Baron A, Renard CMGC, Péan C, Drilleau J-F (2000). Different action patterns for apple pectin methylesterase at pH 7.0 and 4.5. Carbohydr. Res..

[14] Savary BJ, Hotchkiss AT, Cameron RG (2002). Characterization of a salt-independent pectin methylesterase purified from Valencia orange peel. J. Agric. Food Chem..

[15] Cameron RG, Luzio GA, Goodner K, Williams MAK (2008). Demethylation of a model homogalacturonan with a salt-independent pectin methylesterase from citrus: I. Effect of pH on demethylated block size, block number and enzyme mode of action. Carbohydr. Polym..

[16] Fry, S.C. Plant cell wall polymers in *Biofuels and Bioenergy*, edited by Love, J. and Bryant, J.A., pp. 59–87 (John Wiley & Sons Ltd, 2017).

[17] Videcoq P, Garnier C, Robert P, Bonnin E (2011). Influence of calcium on pectin methylesterase behaviour in the presence of medium methylated pectins. Carbohydr. Polym..

[18] Videcoq P, Steenkeste K, Bonnin E, Garnier C (2013). Multi-scale study of enzyme diffusion in macromolecular solutions and physical gels of pectin polysaccharides. Soft Matter.

[19] Vincent RR, Cucheval A, Hemar Y, Williams MAK (2009). Bio-inspired network optimization in soft materials — Insights from the plant cell wall. Eur. Phys. J. E.

[20] Bonnin E (2015). Methylesterase behaviour is related to polysaccharide organisation in model systems mimicking cell walls. Carbohydr. Polym..

[21] Agoda-Tandjawa G, Durand S, Gaillard C, Garnier C, Doublier J-L (2012). Rheological behaviour and microstructure of microfibrillated cellulose suspensions/low-methoxyl pectin mixed systems. Effect of calcium ions. Carbohydr. Polym..

[22] Jamme F (2010). Synchrotron UV Fluorescence microscopy uncovers new probes in cells and tissues. Microsc. Microanal..

[23] Jamme F (2013). Deep UV autofluorescence microscopy for cell biology and tissue histology. Biol. Cell.

[24] Tawil G (2011). *In situ* tracking of enzymatic breakdown of starch granules by Synchrotron UV fluorescence microscopy. Anal. Chem..

[25] Devaux M-F (2018). Synchrotron time-lapse imaging of lignocellulosic biomass hydrolysis: tracking enzyme localization by protein autofluorescence and biochemical modification of cell walls by microfluidic infrared microspectroscopy. Front. Plant Sci..

[26] Agoda-Tandjawa G (2010). Rheological characterization of microfibrillated cellulose suspensions after freezing. Carbohydr. Polym..

[27] Christgau S (1996). Pectin methylesterase from *Aspergillus aculeatus*: expression cloning in yeast and characterization of the recombinant enzyme. Biochem. J..

[28] Giuliani A (2009). DISCO: a low-energy multipurpose beamline at synchrotron SOLEIL. J Synchrotron Radiation.

[29] Tomazevic D, Likar B, Pernus F (2002). Comparative evaluation of retrospective shading correction methods. J. Microsc..

[30] Soille, P. Morphological image analysis in *Principles and applications, 2nd ed*. (Springer-Verlag, New-York, 2003).

[31] Lin D, Lopez-Sanchez P, Gidley M-J (2016). Interactions of pectins with cellulose during its synthesis in the absence of calcium. Food Hydrocoll..

[32] Agoda-Tandjawa G, Durand S, Gaillard C, Doublier J-L (2012). Properties of cellulose/pectins composites: Implication for structural and mechanical properties of cell wall. Carbohydr. Polym..

[33] Amsden B (1998). Solute diffusion within hydrogels. Mechanisms and Models. Macromolecules.

[34] Masaro L, Zhu XX (1999). Physical models of diffusion for polymer solution, gels and solids. Progr Polym Sci.

[35] Assifaoui A (2015). Structural behaviour differences in low methoxy pectin solutions in the presence of divalent cations (Ca^2+^ and Zn^2+^): a process driven by the binding mechanism of the cation with the galacturonate unit. Soft Matter.

[36] Paës G (2013). Modeling progression of fluorescent probes in bioinspired cellulosic assemblies. Biomacromolecules.

[37] Couret L, Irle M, Belloncle C, Cathala B (2017). Extraction and characterization of cellulose nanocrystals from post-consumer wood fiberboard waste. Cellulose.

[38] Wilkins DK (1999). Hydrodynamic radii of native and denatured protein measured by pulse field gradient nmr techniques. Biochem..

[39] Jee A-Y, Cho Y-K, Granick S, Tlusty T (2018). Catalytic enzymes are active matter. Proc. Nat. Acad. Sci. USA.

[40] Riedel C (2015). The heat released during catalytic turnover enhances the diffusion of an enzyme. Nature.

[41] Sénéchal F, Wattier C, Rustérucci C, Pelloux J (2014). Homogalacturonan-modifying enzymes: structure, expression, and roles in plants. J. Exp. Bot..

[42] Ding S-Y (2012). How does plant cell wall nanoscale architecture correlate with enzymatic digestibility?. Science.

[43] Luterbacher JS, Moran-Mirabal JM, Burkholder EW, Walker LP (2015). Modeling enzymatic hydrolysis of lignocellulosic substrates using fluorescent confocal microscopy II: pretreated biomass. Biotechnol. Bioeng..

[44] Donaldson L, Vaidya A (2017). Visualising recalcitrance by colocalisation of cellulase, lignin and cellulose in pretreated pine biomass using fluorescence microscopy. Sci. Rep..

