# Supplementary material for: Mobility of pectin methylesterase in pectin/cellulose gels is enhanced by the presence of cellulose and by its catalytic capacity
Source: Sci Rep. 2019 Aug 29;9:12551. doi: 10.1038/s41598-019-49108-x (PMC6715659; doi:10.1038/s41598-019-49108-x)
Supplement: Supplementary file 1 — Supplementary dataset 1 [file 41598_2019_49108_MOESM1_ESM.pdf]

Mobility of pectin methylesterase in pectin/cellulose gels is enhanced by the presence of cellulose and by its catalytic capacity

Estelle Bonnin, Camille Alvarado, Marie-Jeanne Crépeau, Brigitte Bouchet, Catherine Garnier, Frédéric Jamme, Marie-Françoise Devaux

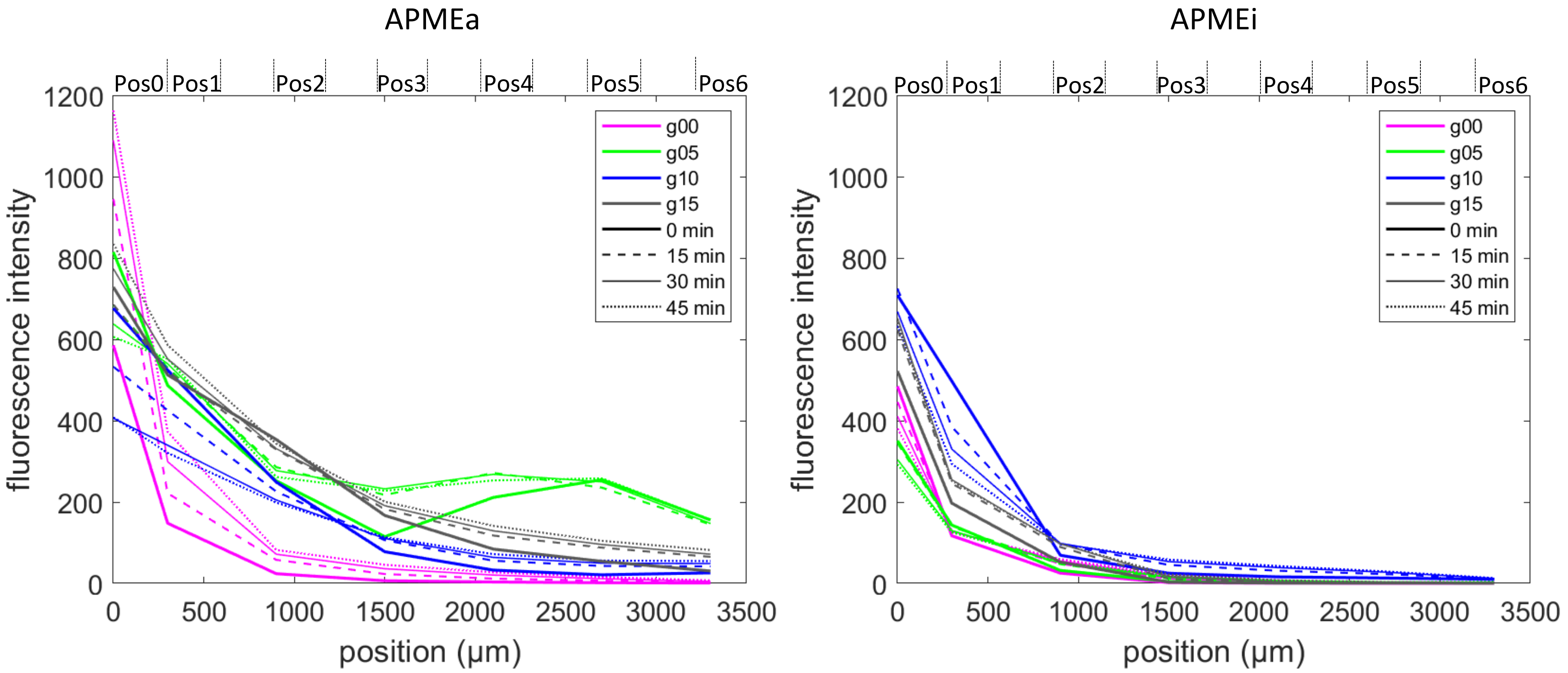

Supplementary data: Evolution of fluorescence intensity as a function of Position. The fluorescent intensities were extracted from images acquired on all positions at 0, 15, 30 and 45 min for the native (APMEa) and the heat-inactivated (APMEi) enzyme and plotted as a function of position without normalisation. The gels were prepared by mixing 1.5% LM pectin with no cellulose (g00, pink), 0.5% cellulose (g05, green), 1.0% cellulose (g10, blue), 1.5% cellulose (g15, black).
